# Editorial: Methods and applications in psychopathology: new methods and trends for the understanding of neuropsychiatric disorders

**DOI:** 10.3389/fpsyg.2023.1242921

**Published:** 2023-07-20

**Authors:** Chong Chen, Gabriele Nibbio, Yuka Kotozaki

**Affiliations:** ^1^Division of Neuropsychiatry, Department of Neuroscience, Yamaguchi University Graduate School of Medicine, Ube, Japan; ^2^Department of Clinical and Experimental Sciences, University of Brescia, Brescia, Italy; ^3^Department of Hygiene and Preventive Medicine, School of Medicine, Iwate Medical University, Morioka, Japan

**Keywords:** psychopathology, depressive disorders, anxiety disorders, psychiatric diseases, computational psychiatry, data-driven approach, probability weighting, latent-class analysis

Neuropsychiatric disorders are highly prevalent and are causing an enormous disease burden globally. For instance, according to an estimation by the World Health Organization, 4.4 and 3.6% of the global population lived with depressive and anxiety disorders in 2015, respectively (World Health Organization, 2017). The Global Burden of Diseases, Injuries, and Risk Factors Study estimated that in 2019, mental disorders accounted for 4.9% of global disability-adjusted life-years (DALYS), making it one of the top ten leading causes of disease burden globally (GBD 2019 Mental Disorders Collaborators, 2022). One of the most important causes of such a high prevalence, high disability of neuropsychiatric disorders is our lack of precise understanding of the psychopathological and neurobiological mechanisms underlying these disorders, which holds us back from developing effective prevention and treatment strategies.

This Research Topic is a collection of eleven cutting-edge studies that help advance our understanding of the psychopathological and neurobiological mechanisms of neuropsychiatric disorders with novel methods and techniques. Specifically, six studies take a computational psychiatry approach. One study takes a neuroimaging approach, one genetic approach, and one psychometrical approach. Finally, two studies take a meta-analytic approach.

Computational psychiatry represents a novel approach that tries to solve problems in psychiatry by applying mathematical methods (Montague et al., 2012; Chen et al., 2015; Friston, 2023). One theory-driven approach attempts to provide mechanistic insights by specifying and testing psychological and neurobiological processes that are believed to generate the illness or its symptoms. This approach is in line with current conceptualization of mental disorders based on neurobiological processes such as the Research Domain Criteria (RDoC) project (Insel et al., 2010; Barlati et al., 2020) and the Hierarchical Taxonomy of Psychopathology (HiTOP) (Kotov et al., 2022). Another data-driven approach attempts to make predictions of diagnosis or treatment with a theory-free perspective by employing various statistical models and machine learning algorithms. Six articles in this Research Topic employ either a theory- or data-driven approach.

Soda et al. provides mechanistic insights into developmental disorders with a computational model that combines hierarchical Bayesian models and neural network models. In this model, the agent learns the underlying probabilistic structure of the environment with a neural system that acquires a hierarchical Bayesian representation of the underlying structure. In their simulation, the authors manipulated the neural stochasticity in the environment during learning and found that when the neural stochasticity is low (meaning that neural noise is low and that the agent's behavior tends to be deterministic), the agent showed altered hierarchical representation and reduced flexibility, mimicking cognitive changes in developmental disorders. Gauld and Depannemaecker proposes a generic model aimed at capturing the dynamics of psychiatric symptoms. In this model, the symptoms of psychiatric diseases are simulated with internal factors, temporal specificities and symptomatology, with environmental noises. With this model, the authors simulated three different psychiatric disorders. Schizophrenia is characterized by pathology evolving following an outbreak; bipolar disorders is characterized by kindling and bursts in symptoms; persistent complex bereavement disorder is characterized by susceptibility to the external environment (i.e., bereavement).

Hagiwara et al. tries to clarify the common vs. unique cognitive computational mechanisms associated with depression and anxiety, two disorders that often co-occur (Chen, 2022). According to the Cumulative Prospect Theory (Tversky and Kahneman, 1992), people tend to overweight small probabilities and underweight large probabilities (i.e., an inverted S-shaped nonlinear probability weighting curve). Hagiwara et al. found that subjects with more depressive but not anxiety symptoms tend to show a weakened inverted S-shaped probability weighting curve, indicating increased risk aversion at small probabilities and reduced risk aversion at large probabilities. Such an alteration in probability weighting has also been reported in females under high levels of chronic stress (Lei et al., 2021). Interestingly, two studies (Shimizu et al.; Watarai et al., 2023) demonstrate that recalling positive autobiographical memories, such as memories of a happy family vacation, affects probability weighting in a way that is in the opposite direction compared to the influence by depressive symptoms (Hagiwara et al.) and chronic stress in females (Lei et al., 2021). These findings suggest the potential usefulness of such an intervention for the treatment of altered decision-making in psychiatric disorders.

Lei et al. advances our understanding of the comorbidity of depressive and anxiety disorders by investigating the patterns of the co-occurrence of their symptoms. Using latent class analysis, a data-driven approach, the authors identified four symptoms patterns or classes that are characterized by different levels of co-occurrence of depressive and anxiety symptoms. Furthermore, each pattern is associated with distinct psychological risk factors, indicating unique etiologies. Chacko et al. provides insights into the link of stress to PTSD and obesity using natural language processing of over 10,000 peer-reviewed publications. The authors identified 34 metabolic mediators, among which Neuropeptide-Y and cortisol were found to reduce PTSD severity but worsen obesity, while oxytocin reduces both PTSD severity and obesity.

Cognitive task-based neuroimaging such as functional near-infrared spectroscopy is another useful paradigm for the study of neuropsychiatric diseases. In a perspective article, Ren et al. gives a comprehensive introduction to a multi-cognitive task paradigm that includes emotional picture identification task, verbal fluency task, and so on. By alternating between resting state and different tasks, the authors argue, this paradigm helps to probe the function of different brain networks in neuropsychiatric diseases.

Rohlfing et al. employs a genetic approach and investigates the association between catechol-O-methyltransferase (COMT) Val158Met polymorphism and substance use. COMT is an enzyme that degrades catecholamines and the presence of the Met allele in the COMT Val158Met polymorphism is associated with lower COMT activity and thus higher concentrations of extra-synaptic dopamine. The authors found that the presence of the Met allele is associated more cigarettes smoked per day and lower motivation on the monetary incentive delay task. Li et al. takes a meta-analytic approach and evaluates the effect of cognitive bias modification (CBM) on depressive symptoms in adults. CBM is a therapy grounded in the Cognitive Theory of depression which proposes that cognitive bias underlies the development and maintenance of depressive symptoms by changing the availability of negative vs. positive information (Beck and Dozois, 2011). CBM, on the other hand, aims to change these cognitive biases. Based on ten randomized controlled trials, Li et al. reported a significant effect of CBM on depressive symptoms with g = −0.64. Moreover, the effect size is higher for CBM targeting interpretation (g = −1.45) compared to targeting attention (g = −0.63) or imagery (g = −0.48). Khan et al. also takes a meta-analytic approach and estimates the prevalence of neuropsychiatric symptoms in patients with systemic lupus erythematosus (SLE) in Pakistan. Based on thirteen studies, the authors estimate that the prevalence is the highest for cognitive dysfunction (32%), followed by headache (10%), seizures (6%), and psychosis (4%). The high prevalence emphasizes the importance of effective management of neuropsychiatric symptoms in addition to clinical treatment of SLE itself. Lastly, Rakshasa-Loots and Laughton employs a psychometric approach and presents the protocol designed to test the reliability and validity of the isiXhosa Translation of the Patient Health Questionnaire (PHQ-9), a widely employed measure of depression severity.

## Author contributions

Manuscript drafting: CC. Manuscript revising and approval: All authors. All authors contributed to the article and approved the submitted version.
